# Gene regulation knowledge commons: community action takes care of DNA binding transcription factors

**DOI:** 10.1093/database/baw088

**Published:** 2016-06-05

**Authors:** Sushil Tripathi, Steven Vercruysse, Konika Chawla, Karen R. Christie, Judith A. Blake, Rachael P. Huntley, Sandra Orchard, Henning Hermjakob, Liv Thommesen, Astrid Lægreid, Martin Kuiper

**Affiliations:** ^1^Department of Cancer Research and Molecular Medicine, Norwegian University of Science and Technology (NTNU), 7491 Trondheim, Norway; ^2^Department of Biology, Norwegian University of Science and Technology (NTNU), 7491 Trondheim, Norway; ^3^Department of Computational Biology and Bioinformatics, The Jackson Laboratory, 600 Main Street, Bar Harbor, ME, USA; ^4^Centre for Cardiovascular Genetics, Institute of Cardiovascular Science University College, London WC1E 6JF, UK; ^5^European Molecular Biology Laboratory, European Bioinformatics Institute (EMBL-EBI), Wellcome Trust Genome Campus, Hinxton, Cambridge CB10 1SD, UK; ^6^Department of Medical Laboratory Technology, Norwegian University of Science and Technology (NTNU) 7491 Trondheim, Norway

## Abstract

A large gap remains between the amount of knowledge in scientific literature and the fraction that gets curated into standardized databases, despite many curation initiatives. Yet the availability of comprehensive knowledge in databases is crucial for exploiting existing background knowledge, both for designing follow-up experiments and for interpreting new experimental data. Structured resources also underpin the computational integration and modeling of regulatory pathways, which further aids our understanding of regulatory dynamics. We argue how cooperation between the scientific community and professional curators can increase the capacity of capturing precise knowledge from literature. We demonstrate this with a project in which we mobilize biological domain experts who curate large amounts of DNA binding transcription factors, and show that they, although new to the field of curation, can make valuable contributions by harvesting reported knowledge from scientific papers. Such community curation can enhance the scientific epistemic process.

Database URL: http://www.tfcheckpoint.org

## Introduction

We call for the broader Life Sciences community to engage in knowledge curation: to compile knowledge from literature into well-structured formats. We show this by example, with our own efforts in curating the scientific literature for knowledge about DNA binding transcription factors (DbTFs) in the model species human, mouse and rat. DbTFs guide the RNA polymerase II transcription machinery to specific gene regulatory elements, and play a crucial role in the targeted unlocking of information in the genome. According to bioinformatics analyses, the genome-scale repertoire of DbTFs in humans may comprise around 1700–1800 proteins (1, 2). We found that half of these appear to have been experimentally studied and validated (3), yet only a fraction of these have been entered into databases together with sufficient details on biological context and adequate experimental validation. Thus, much of this knowledge remains hidden in the scientific literature. As archiving this knowledge into appropriate databases can only be achieved through dedicated human cognition, a community effort is needed in *curation*: the taking care of knowledge.

Although many ongoing professional curation projects exist (4–7), it is believed that they cannot keep up with the increasing flow of scientific publications (8). Evidently we need to explore new strategies, and we argue that curation is also possible with closer involvement of non-professional curators or indeed the scientific community as a whole. We previously (9) proposed a set of curation guidelines that individuals of the scientific community can apply to curate DbTF knowledge, and herewith enrich existing and well-maintained knowledge bases such as the Gene Ontology (GO) database (10) and UniProt (5). Such community curation is valuable for ongoing research in several ways: (i) it feeds into a comprehensive resource of background knowledge, essential for computational analysis and the design of new experiments in an informed way; (ii) it makes one carefully consider what type of experimental evidence is necessary and sufficient to support assertions, in our case, the functional annotation of a DbTF; and (iii) it creates an overview on those proteins among the current DbTF candidates that still lack proper evidence, and therefore should be subjected to intensified small- and large-scale experimental efforts, as discussed in Ref. (11), to complete their characterization.

This paper reports on an initiative of a group of domain-expert scientists teaming up with professional curators, to exhaustively curate experimental evidence about DbTFs from human, mouse and rat. This effort generates enhanced resources that will provide unique, computationally accessible data about mammalian transcription factors for the research community and will thereby boost genome-wide understanding of gene regulation. This result demonstrates that community curation can make a difference.

## DbTF knowledge today is spread over disparate and largely incomplete resources

**Figure 1.**
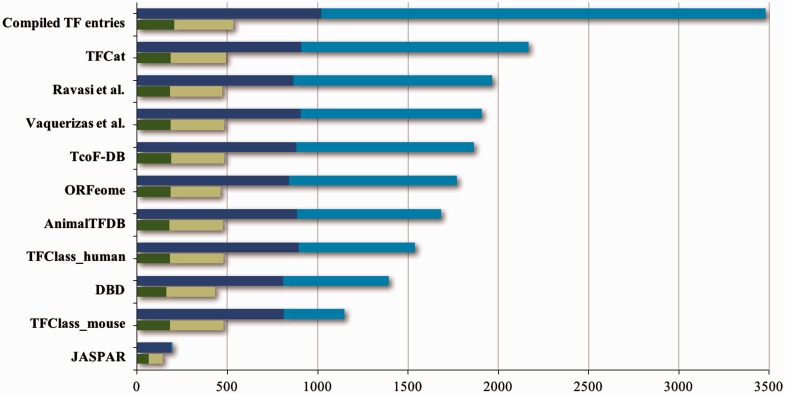
Contents of TF resources. For each TF database resource two bars are shown: the total number of unique entries is indicated by blue bars, the dark blue part of which indicates specific DNA binding transcription factors (DbTFs) for which we have found literature evidence (3). The green bars below each blue bar represent the numbers of DbTFs present within that resource that are corroborated in the GO database by annotation with experimental evidence to the GO term GO:0000981, or child terms thereof. Dark green: DbTFs documented in the GO database at the start of our project March 2013 (205); Light green: new entries after March 2013 (328). Numbers in parentheses give the cumulative total in TFcheckpoint and refer to human, mouse or rat DbTFs, with orthologues counted only once. Of the 328 new experimentally documented DbTF annotations (light green), 301 were uniquely provided by our current project. The GO database version referenced here, which includes our new annotations, is dated 06 December 2014. Data versions for the other sources are given at www.tfcheckpoint.org.

**Figure 2.**
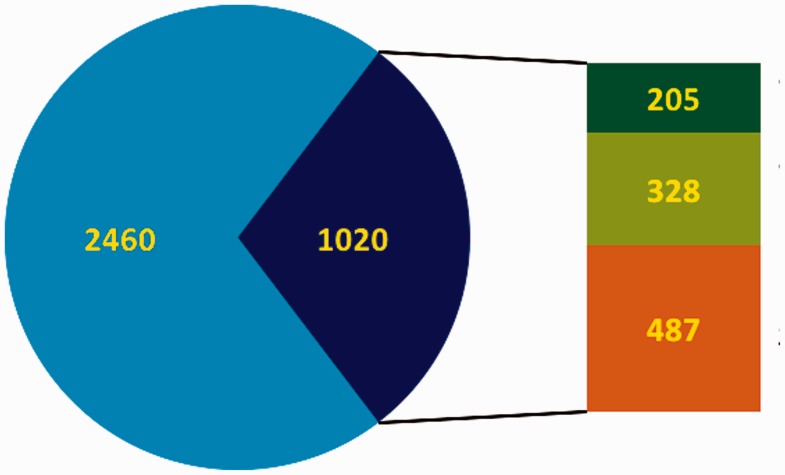
Overview of the curation status of DbTFs. In the pie chart blue represents the total number of candidate TFs, and the dark blue part indicates DbTFs with literature reference (3). Note that only 1700–1800 of the candidate TFs (blue) are considered DbTFs (1, 2). In the bar to the right of the pie part green represents the number of curated DbTFs in the GO database (dark green: before March 2013, light green: after March 2013 when we started our community curation efforts. Orange indicates the number of DbTFs with literature reference (3) that still need to be curated.

A considerable number of transcription factor databases and resources have been compiled, all providing structured information about transcription factors (Table 1). However, many of these resources do not provide standardized or verifiable experimental evidence that would reflect the level of support for these proteins’ functional role annotations. As an exception, the GO database (10) does provide high quality descriptions and evidence both for the DNA-binding and the RNAPII regulatory functions of DbTFs, by way of annotations with the GO term *sequence-specific DNA binding RNA polymerase II transcription factor activity* (GO:0000981), or terms that are even more specific. The IntAct database in addition supports recording of the target genes experimentally shown to be regulated by a particular DbTF (12). As a further illustration of the diversity and spread of the information from 10 prominent transcription factor resources, their combination and alignment of orthologous proteins shows that together they list almost 3500 unique protein entries (ortholog groups) (Figure 1) for human, mouse and rat. Noticeably, most of the transcription factor resources (Table 1) do not distinguish well between true DbTFs and other transcription regulators, like factors that act through protein interactions or chromatin modifications. Exceptions are TFClass (2), AnimalTFDB (13), TFCat (14), the GO database (10) and IntAct (12). Further analysis of listed proteins against the literature indicates that about 1000 of the ortholog groups have at least one member with some form of experimental evidence that would support that they indeed may be qualified as DbTFs (Figure 1). However, only 205 of these were fully annotated in the GO database at the start of our project (Figures 1 and 2).

**Table 1.** Overview of resources for mammalian transcription factors

Resources	Description	Entries*	URL/PMID
AnimalTFDB	Animal transcription factor database	1682	http://www.bioguo.org/AnimalTFDB/
CIS-BP	Determination and inference of eukaryotic transcription factor sequence specificity	1017	http://cisbp.ccbr.utoronto.ca/
DBD	Database of predicted transcription factors in completely sequenced genomes	1395	http://www.transcriptionfactor.org
footprintDB	Database of transcription factors with annotated cis elements and binding interfaces	2422	http://floresta.eead.csic.es/footprintdb
GO database	Community-based bioinformatics resource that classifies gene product function through the use of structured, controlled vocabularies	1121**	http://geneontology.org/page/go-database
HOCOMOCO	Comprehensive collection of human transcription factor binding sites models	601	http://hocomoco.autosome.ru
HTRIdb	Repository of experimentally verified interactions among human TFs and their respective target genes	284	http://www.lbbc.ibb.unesp.br/htri/
IntAct	Molecular interaction database populated by data either curated from the literature or from direct data depositions	607***	http://www.ebi.ac.uk/intact
JASPAR	Matrix-based nucleotide profiles describing the binding preference of transcription factors from multiple species	202	http://jaspar.genereg.net
PAZAR	Transcription factor and regulatory sequence annotation. Unites independently created and maintained data collections	708	http://www.pazar.info
TcoF-DB	Human transcription co-factors and transcription factor interacting proteins.	1864	http://cbrc.kaust.edu.sa/tcof
TFCat	Mouse and human TFs based on a reliable core collection of annotations obtained by expert review of the scientific literature database	1052	http://www.tfcat.ca
TFcheckpoint	Curated compendium of specific DNA-binding RNA polymerase II transcription factors	3480	http://www.tfcheckpoint.org
TFClass	Classification of human transcription factors and their rodent orthologs	1558	http://tfclass.bioinf.med.uni-goettingen.de/tfclass
TFe	Compendium of mini review articles on transcription factors (TFs) that is founded on the principles of open access and collaboration	803	http://cisreg.cmmt.ubc.ca/cgi-bin/tfe/home.pl
TRANSFAC	Transcription factors, their binding sites, nucleotide distribution matrices and regulated genes	1040	http://www.gene-regulation.com/pub/databases.html
TRED	Transcriptional Regulatory Element Database and a platform for in silico gene regulation studies	36	https://cb.utdallas.edu/cgi-bin/TRED/tred.cgi?process=home
Jolma et al.	DNA-binding specificities of human transcription factors	411	PMID: 23332764
Messina et al.	ORFeome-based analysis of human transcription factor genes	1770	PMID: 15489324
Ravasi et al.	Atlas of combinatorial transcriptional regulation in mouse and man; physical interactions among the majority of human and mouse DNA-binding transcription factors	1967	PMID: 20211142
TFCONES	Vertebrate transcription factor-encoding genes and their associated conserved non-coding elements. Content integrated with AnimalTFDB.	1962	PMID: 18045502
Vaquerizas et al.	Census of human transcription factors: function, expression and evolution; analysis of 1391 manually curated sequence-specific DNA-binding transcription factors, their functions, genomic organization and evolutionary conservation	1909	PMID: 19274049

Contents of the individual resources are summarized with a brief description and number of entries. Link to each of the resources are also provided as URL or PMID.

* Numbers obtained from these resources on 3 March 2016.

** Entries annotated with GO term GO:0003700 or more specific.

*** Interactions where A is a protein annotated to GO0000981 (or child thereof) and B is a gene

## Progress and initial results of our community effort

To take on the challenge of curating the remaining literature and archiving this knowledge into databases, a group of domain experts at the Norwegian University of Science and Technology (NTNU) teamed up with professional curators at the Gene Ontology Consortium (GOC) and at the European Bioinformatics Institute (EMBL-EBI). Our initiative builds upon NTNU’s earlier work with TFcheckpoint (http://www.tfcheckpoint.org) and is enabled by the web app SciCura (http://scicura.org, to be published elsewhere). Its curation results are exported to both GOC’s GO database (http://www.geneontology.org) and EMBL-EBI’s IntAct database (http://www.ebi.ac.uk/intact/).

Together we work towards three aims: (i) Protocol: we developed a detailed protocol for identifying, characterizing and qualifying knowledge about the DNA-binding and RNAPII regulatory functions of DbTFs in the scientific literature and made the protocol publicly available to serve as curation guidelines (9); (ii) Survey: we have used these guidelines to survey the scientific literature and retrieve those human, mouse and rat proteins that are reported as having sequence-specific DNA binding transcription factor activity; and (iii) Annotation: we are in the process to carefully check the experimental evidence and—where fully substantiated—annotate these proteins with the appropriate DbTF GO terms, and submit these new annotations to the GO database. The GO database in total has 1121 unique entries (Table 1) with TF-related terms (release 6 December 2014), and our community curation effort has so far resulted in TF-relevant annotations for a total of 379 human, mouse or rat proteins in the GO database. Among these are 328 new DbTF annotations to GO:0000981, or child terms thereof. Combined with annotations contributed by others, the total number of experimentally documented unique DbTFs (human, mouse and rat ortholog groups) now available in the GO database is 533. Thus, our community-based effort more than doubled the number of DbTF annotations in the GO database (Figures 1 and 2).

Our current aim is to complete the curation task for the remaining ∼500 human, mouse and rat DbTFs (Figure 2) for which some form of experimental evidence could be found in papers referenced in the original resources or in other published papers. Our curation procedure captures a specific level of detail since we annotate DbTFs together with the experimental context in which they were assessed. Whenever possible, we also annotate to which specific target genes or nucleic acid sequences the DbTFs bind and will feed this information into the IntAct molecular interaction database (12) and into the GO database through the ‘Annotation Extension’ field (15). The PSI-MI controlled vocabulary (3) supports such detail through a wide range of terms on experimental setting and DbTF interaction with target genes and other transcription regulators. For example, we already identified over 400 DbTF:target gene interactions that were documented with electrophoretic mobility shift assays (EMSA), described in over 170 different scientific papers. Many interactions appear in various papers and experiment types, and we create many additional annotations accordingly. Additional information, such as whether the binding resulted in an up- or down-regulation of the gene in this cell/tissue type, under the described experimental conditions, has also been curated into the database.

Our joint work essentially mobilizes ‘dormant’ knowledge. It gives the scientific community much needed access (16) to high quality and exhaustive information through central resources, and so accommodates many aspects of the scientific discovery process, among others rapid progress in genome annotation. Hosting this knowledge in well-established databases like GO and IntAct has several advantages: (i) the knowledge becomes available to all analysis approaches [both manual and (semi-)automated] that use GO annotations or IntAct interaction data; (ii) these databases impose essential standards that warrant quality and consistency across different annotations, for instance when community curators use web-based curation tools developed by and for these major resources; and (iii) this knowledge is maintained and regularly synchronized with changes to the underlying reference sequence databases and controlled vocabularies, with computational pipelines already established for these databases.

## Confidence-scored annotations facilitate ranking of gene regulation hypotheses

The added value of specifying the experimental context in which functional evidence was obtained should not be underestimated. Such experimental details can be specified with PSI-MI terms, for instance for DNA binding. This enables confidence scoring in a manner analogous to the MIscore protocol established for protein–protein interactions (17), available through the PSISCORE registry (18). Confidence measures enable scientists to utilize *all* available functional annotations, regardless of the level of experimental support. Particular subsets of DbTFs or DbTF:target gene interactions can then be chosen, depending on how stringent the supporting evidence must be for a particular use case. For example, regulatory network building would often take into account only DbTF:target gene interactions meeting the highest confidence criteria, whereas the integration of genome-scale data sets for high-throughput hypothesis assessment may consider interactions supported by any confidence level. In this context, a central challenge is to provide full transparency of the suggested scoring criteria, and also to provide access to the detailed underlying evidence in a way that enables users to implement their own scoring or selection criteria. For example: IntAct records the specific version of an EMSA experiment used for establishing interaction of DbTFs and target DNA sequences. This allows a user to select only annotations based on high-confidence EMSAs that use purified DbTF protein, and to dismiss EMSA experiments performed with nuclear extracts, as the latter leave open the possibility that proteins other than the putative DbTF mediated DNA-binding.

Our work also leads us to contribute to the PSI-MI vocabulary. While we curate, we encounter opportunities to refine and extend the PSI-MI vocabulary with terms that allow for a more differentiated annotation of experimental evidence, and for documenting causal, transcription regulatory relations between DbTFs and their target genes. Work like this will further increase the power of scoring opportunities and the rich semantic depth of structured knowledge.

## Future prospects

Much work remains in harvesting valuable information and enabling knowledge from the scientific literature, not only about DbTFs but also protein-binding transcription regulators, chromatin modulators, etc., or indeed proteins and other biological components in any other biological domain. The experts most qualified for this task are out there, in the scientific community. All of us can significantly complement professional programs; participate in the development of curation protocols, ontologies, and annotation databases; and allow colleagues to benefit from cooperative efforts like the one described here.

We encourage funding agencies to acknowledge our shared responsibility for taking care of knowledge generated in costly research activities, as current practices may lead to waste: discovered knowledge, or sometimes re-discovered knowledge which is not made commonly available in a format easily enabling computational retrieval. We hope that the scientific community as a whole can identify incentives and place increasing emphasis on various important curation endeavors. This includes continuing to support the professional curation programs that guarantee the necessary foundation for data governance, maintenance of standards, databases, access through web-interfaces and automated data exchange technologies. Only then can valuable results of public financing persist, become broadly available in formats practical for consumption, and increase the general efficacy of research projects. In addition, we call for the scientific community to explore new approaches for ‘curation at the source’. Perhaps efforts are needed for lowering thresholds to curation and for persuading or rewarding (19) the original authors of a paper, the ultimate domain experts, to perform curation of their findings as a final pre-publication step?

## Conclusions

We are confident that the product of curation, including efforts described here, will serve as a reference for both small-scale assembly of regulatory pathways, and genome-scale analyses of gene regulatory networks [such as ENCODE for genome-scale DbTF function evidence (20)]. Our curation approach creates a thorough overview of what we know, and appreciates the experimental detail and rigor necessary to be confident about what we know. This is essential for launching effective new initiatives to characterize biological components and their interactions, and necessary for building detailed system-wide gene regulatory network models. Such models provide the molecular mechanistic scaffolds that can support not only fundamental research, but also systems medicine and targeted, higher precision health care.

Our story provides evidence that joint action can make a difference. We learned that a community of volunteering domain experts can team up with professional curators and together develop specific and effective curation protocols. A community can more readily identify gaps and hurdles in ontologies that are needed to capture essential experimental context and biological relationships. A community can together make a significant impact on the information available from annotation databases. The impact of such actions grows larger as more of our responsible colleagues step up and mobilize their peers to take similar action. We welcome colleagues to get into contact, as together we can share and evolve the procedures and tools to get additional efforts accomplished.

## Authors contributions

ST conceived the idea, participated in DbTF annotations, their quality check and manuscript writing. SV designed and developed a web app SciCura that enables community curation of DbTFs at NTNU and participated in manuscript writing. KC maintained the TFcheckpoint database and helped to coordinate the curation process. KRC, JAB participated in mouse DbTF annotations, quality check and manuscript writing. RPH participated in human DbTF annotations, quality check, submission to GO database and manuscript writing. SO, HH participated in DbTF - target gene annotation, submission to the IntAct database and manuscript writing. LT participated in DbTF annotation and their quality check. MK, AL supervised idea conception, participated in DbTF annotation, their quality check and manuscript writing. All authors read and approved the final manuscript.
